# Influence of Aortoiliac Geometry on Non-Occlusive Thrombotic Risk Following Endovascular Repair of Abdominal Aortic Aneurysms

**DOI:** 10.3390/diagnostics15172134

**Published:** 2025-08-24

**Authors:** Jeong In Lee, Dac Hong An Ngo, Hong Pil Hwang, Young Min Han, Hyo Sung Kwak

**Affiliations:** 1 Department of Medicine, Jeonbuk National University, Jeonju 54907, Republic of Korea; wjddls830@naver.com; 2 Department of Radiology, Research Institute of Clinical Medicine of Jeonbuk National University-Biomedical Research Institute of Jeonbuk National University Hospital, Jeonbuk National University and Medical School, Jeonju 54907, Republic of Korea; ngodachongan@hueuni.edu.vn (D.H.A.N.); ymhan@jbnu.ac.kr (Y.M.H.); 3 Department of Radiology, Hue University of Medicine and Pharmacy, Hue 530000, Vietnam; 4 Department of Surgery, Jeonbuk National University Hospital, Jeonju 54907, Republic of Korea; h2p@jbnu.ac.kr

**Keywords:** geometry, thrombosis, endovascular aneurysm repair, aorta, iliac artery

## Abstract

**Objectives**: This study investigated the impact of aortoiliac geometry on thrombotic complication following aortic endovascular aneurysm repair (EVAR). **Methods**: Data from 54 patients who received abdominal EVAR between January 2015 and December 2023, in which 18 developed unilateral iliac limb in-stent thrombus, were retrospectively reviewed. Clinical data, including cardiovascular risk factors, laboratory findings, and geometrical factors, including iliac diameter, sectional area, limb angles, and tortuosity, were collected. Aortoiliac geometry analyses were performed on the 3D model reconstructed from abdominal aortic computed tomography angiography (CTA) using semi-automated software (MIMICS version 25.0). **Results**: Compared to non-thrombotic limbs, thrombotic iliac limbs showed larger maximum diameters (17.48 ± 0.95 mm vs. 14.14 ± 0.62 mm, *p* = 0.006), lower graft limb angles (117.52° ± 5.61° vs. 148.54° ± 4.31°, *p* < 0.001), lower aortoiliac angles (123.48° ± 4.66° vs. 141.96° ± 4.76°, *p* = 0.009), and higher iliac tortuosities (0.2 ± 0.03 vs. 0.12 ± 0.02, *p* = 0.02). Paired comparisons between normal and diseased limbs in 18 patients with thrombotic events also showed statistical differences in terms of iliac limb maximum diameter, graft limb angle, aortoiliac angle, and iliac tortuosity. **Conclusions**: Thrombosis formation following EVAR in iliac limbs was associated with limb diameter, graft limb angle, aortoiliac angle, and tortuosity.

## 1. Introduction

Endovascular aneurysm repair (EVAR) has become a widely adopted and minimally invasive approach for managing abdominal aortic aneurysms (AAAs) [1]. The procedure entails deploying a stent graft to exclude the aneurysmal sac from systemic circulation, effectively lowering the risk of rupture. Globally, the use of EVAR continues to rise [2,3]. While specific statistics vary by study, recent research has consistently showed that EVAR offers lower mortality and readmission rates compared to open surgery [4,5]. Despite these advantages and improved perioperative and long-term outcomes, EVAR is still associated with several complications. These include endoleaks, migration or collapse of stent grafts, kinking or narrowing of the graft limbs, and graft infections or thromboses, which affect approximately 16% to 30% of patients undergoing the procedure [6]. Thrombus formation, in particular, has been reported in 7.3% to 24% of cases [7,8,9] and is clinically significant due to its potential to cause limb occlusion [10] or distal embolization, both of which carry serious clinical consequences [7]. Given these issues, the present study investigates anatomical features that may influence thrombus development following EVAR.

The geometry of the aortoiliac axis significantly affects hemodynamic flow patterns, which are closely linked to the risk of thrombus formation [11]. Key anatomical features—such as vessel diameter, curvature, and tortuosity—have been found to impact local blood flow characteristics, including areas of stagnant flow, variations in wall shear stress, and platelet activation. These factors are all central to the development of thrombi [12]. Prior research has shown that sharp vessel angulation and increased tortuosity can disturb smooth, laminar flow, leading to zones of flow separation and recirculation. Such disturbed flow conditions create a hemodynamic environment conducive to thrombus formation [12,13].

While numerous studies have investigated how aortic neck anatomy and iliac fixation influence stent migration and endoleaks [14,15,16], the specific geometric factors that contribute to thrombotic events in the iliac limbs following EVAR remain relatively understudied. Understanding these anatomical parameters is essential for both procedural planning and postoperative management, as they can guide device selection, implantation techniques, and follow-up imaging strategies. As mentioned in the latest guidelines of the European Society of Cardiology, anatomical measurements obtained from computed tomography angiography (CTA) or magnetic resonance angiography are crucial in preoperative preparation of EVAR. Analyses of these indexes not only help device selection and customization but also in predicting and preventing complications [17].

This study seeks to clarify the relationship between aortoiliac geometric features and the risk of thrombotic complications in the iliac limbs after EVAR. Using three-dimensional reconstructions from CTA, we conducted a retrospective analysis comparing the anatomical characteristics of patients who experienced in-stent thrombosis with those who did not develop thrombotic events. Our previous study revealed that thrombotic iliac segments had larger vessel dimensions and lower wall shear stress, while no significant difference was found in terms of arterial angles and tortuosity [12]. We hypothesized that certain other geometric traits, such as iliac diameter, graft limb angle, aortoiliac angle, and iliac tortuosity, are linked to an increased likelihood of thrombosis following EVAR. Therefore, in this study, the measurements of certain geometric indices (angles and tortuosity) were modified to better reflect the effect of iliac artery out-flow and thrombosis risk (Table A1).

## 2. Materials and Methods

This research was approved by the hospital’s Institutional Review Board (JUH 2019-10-040, approval date: 10 January 2020). Since it was a retrospective analysis, the need for informed consent was waived. All procedures adhered to the ethical principles outlined in the Declaration of Helsinki and followed the institution’s guidelines.

### 2.1. Patient Demographic

This retrospective study analyzed patient data from a single center, focusing on individuals who received stent grafts for the treatment of abdominal aortic aneurysms through endovascular aneurysm repair (EVAR) between 2015 and 2022. All patients were treated with the Endurant stent graft (Medtronic Vascular, Santa Rosa, CA, USA). Following EVAR, antithrombotic medication was only used based on patients’ comorbidities. A total of 2 patients (3.7%) received anti-platelet therapy and 4 patients (7.4%) received oral antithrombotic therapy. Inclusion criteria: (1) patients received infra-renal stent grafts comprising of one aortic and bilateral iliac limb components, and (2) post-EVAR (within 30 days) and follow-up CTA data were available. Exclusion criteria: (1) stent grafts with no iliac limb components or unilateral component, and (2) low-quality CTA images or no follow-up data. Furthermore, in order to preserve consistency within the diseased group, patients with complete iliac limb occlusion were excluded due to the complex nature of their etiology. A total of 54 patients were enrolled, with 36 showing no iliac thrombus on follow-up images and 18 presenting with iliac thrombotic findings.

### 2.2. CTA Data Acquisition and Interpretation

Aortoiliac CTA was conducted using a dual-source CT scanner (SOMATOM Definition Flash; Siemens Healthcare, Erlangen, Germany). Contrast agent (Ultravist; Schering, Berlin, Germany) was administered at 5 mL/sec, followed by saline flush of 50 mL. Imaging parameters included a current of 260–300 mA, 1.0 mm slice thickness for reconstruction, and 5 mm maximum intensity projection (MIP) reconstructions.

**Figure 1:**
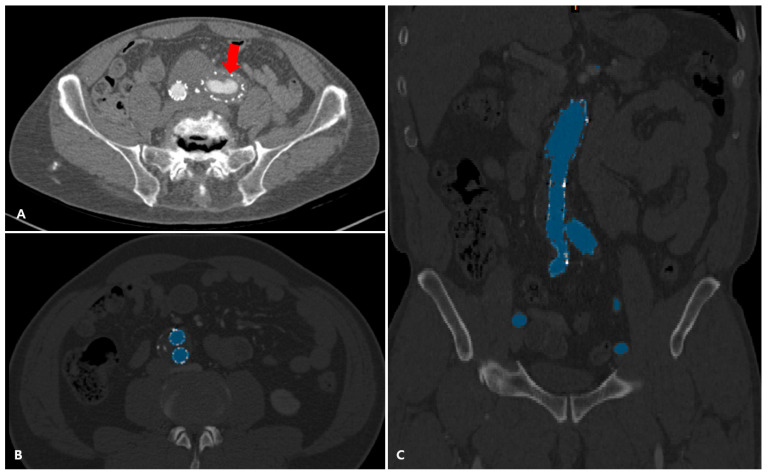
(**A**) Computed tomography angiography (CTA) images of patients with a crescent-shaped thrombus (arrow) of the stent graft iliac component. The 3D model of abdominal aorta to common iliac artery bifurcation was segmented from CTA images (**B**,**C**) using semi-automated software (MIMICS, version 25.0).

Two radiologists, each with over 10 years of experience in vascular imaging and blinded to patient information, independently reviewed all CTA scans. Thrombotic findings were defined by the detection of in-stent thrombus within the iliac component. These appeared as linear or irregular low-attenuation areas inside the stent lumen with a thickness greater than 1 mm and occupying more than 25% of the iliac artery’s circumference (Figure 1A). Patients presenting with thrombus in the aorta but not in the iliac arteries were excluded from the thrombotic group.

### 2.3. Geometry Reconstruction and Measurement

**Figure 2:**
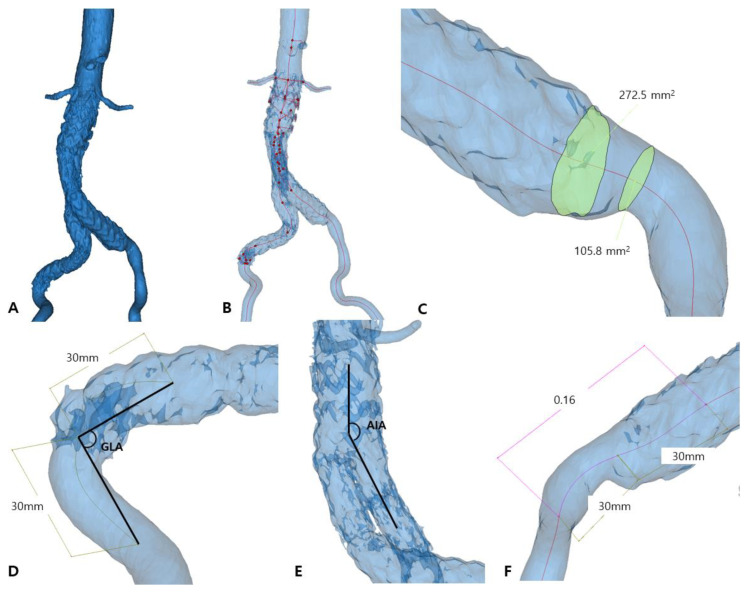
The completed aortoiliac 3D model (**A**) was used to generate the centerline (**B**) and measure cross-sectional area (**C**), graft limb angle (GLA) (**D**), aortoiliac angle (AIA) (**E**), and tortuosity (**F**).

For each patient, data from post-EVAR CTA with no thrombotic findings was retrieved and imported into Materialise MIMICS software (version 25.0; Materialise NV, Leuven, Belgium) for three-dimensional geometric reconstruction. The segmentation procedure, including thresholding, manual segment adjustment, aorta cropping, smart fill, and manual corrections, was performed to construct an aortoiliac lumen 3D model (Figure 1B,C). The model extended from 3 cm above the proximal stent graft to the common iliac artery bifurcation. Smaller arterial branches were removed. A smoothing algorithm was used to enhance the surface of the reconstructed model (Figure 2A).

All geometric analyses were performed in MIMICS on the aortoiliac 3D model generated. The geometric parameters were modified by comparing them to our previous study to better demonstrate the differences between the thrombus and control groups (Table A1). The centerline from the aorta to bilateral iliac arteries was automatically created as the path connecting the centers of the largest inscribed spheres within the vessel lumen (Figure 2B). At each point of interest, a plane perpendicular to the vessel centerline was generated. Within this plane, the software automatically fitted the largest possible circle entirely within the vessel lumen, and the diameter of this inscribed circle was recorded as the local vessel diameter. The cross-sectional area of vessel lumen was measured on a plane perpendicular to the centerline at the point of interest. All values were filtered to identify maximum and minimum values (Figure 2C).

Three key points were then established on the centerline: the iliac stent endpoint and two reference points located 30 mm above and below this endpoint. The graft limb angle (GLA) was defined as the angle between two lines connecting these reference points and the stent endpoint (Figure 2D). The aortoiliac angle (AIA) was calculated as the angle between the centerlines of the aorta and the corresponding iliac artery (Figure 2E). Graft limb tortuosity (GLT) was quantified by calculating the ratio of the direct distance to the centerline length between two defined reference points (Figure 2F). All measurements were taken based on the centerline.

### 2.4. Statistical Analysis

Variables following a normal distribution were reported as mean ± standard deviation, whereas those not normally distributed were presented as median (range). To assess differences in means between the control and thrombus groups, independent sample t-tests were applied for variables with a normal distribution and the Mann–Whitney U test was applied to variables that did not follow a normal distribution. Paired *t*-tests were performed to compare both sides within patients with iliac thrombus. The relationship between thrombotic status and geometric variables was evaluated using the Spearman correlation coefficient (rho). A *p*-value of less than 0.05 was considered statistically significant. All statistical analyses were performed using SPSS software (version 20.0; IBM, Chicago, IL, USA).

## 3. Results

Based on data from patient medical charts and follow-up CTA, unilateral iliac thrombus was detected in 18 of 54 patients (33.3%). There was no significant difference in age and comorbidities between the control and thrombus group (Table 1). The mean time to thrombotic event diagnosis was 9.9 months, ranging from 2 to 36 months. Average follow-up time was 40.7 (12–96) months. In the diseased group, thrombotic clinical impacts were as follows: reintervention (3, 16.7%), distal embolic event (1, 5.5%), and limb loss (0).

Table 2 shows the analysis of post-EVAR iliac segments. Compared to normal limbs, the diseased iliac limbs had significantly larger maximum diameters (17.48 ± 0.95 mm vs. 14.14 ± 0.62 mm, *p* = 0.006), smaller GLAs (117.52 ± 5.61 vs. 148.54 ± 4.31, *p* < 0.001), smaller AIAs (123.48 ± 4.66 vs. 141.96 ± 4.76, *p* = 0.009), and elevated GLTs (0.2 ± 0.03 vs. 0.12 ± 0.02, *p* = 0.021).

Table 3 shows the paired comparison between the normal and thrombus sides in 18 patients with post-EVAR unilateral iliac thrombosis. The diseased side also showed a significantly larger maximum diameter, smaller GLA, smaller AIA, and larger GLT (*p* < 0.05). Another paired comparison was carried out between the right and left iliac segments in the control group and showed no significant difference between the sides (Table 3).

## 4. Discussion

In our study, we identified a significant correlation between the occurrence of iliac thrombotic complications following EVAR and aortoiliac geometric changes including larger iliac diameter and iliac cross-sectional area, smaller GLA and AIA, and increased graft limb tortuosity. The correlation was further supported by a paired analysis of the normal and affected iliac limbs within each patient, revealing similarly significant differences.

The increased diameter and cross-sectional area of iliac limbs can reduce flow velocity, leading to blood stasis and eventually promoting thrombosis [18]. Furthermore, an enlarged vessel may encourage the development of flow separation and recirculation zones, further contributing to stagnant flow and increasing the likelihood of clot formation [19]. These hemodynamic changes are consistent with Virchow’s triad, particularly highlighting the role of abnormal blood flow, such as turbulence and stasis, as a key factor in the development of thrombosis [20].

Regarding vessel angles, our study found that thrombosis is more likely to occur in vessels with sharper, more acute angulations—specifically, reduced aortoiliac and graft limb angles. Such angulated vessels can disrupt normal, laminar flow and create areas of disturbed flow, which may elevate shear stress on the vessel wall and promote platelet aggregation [21]. Additionally, sharper angles are associated with increased endothelial damage, further contributing to thrombus formation [22]. Previous computational fluid dynamics (CFD) studies have shown that acute angulations at arterial bifurcations lead to changes in wall shear stress gradients that are closely linked to endothelial injury and the onset of thrombosis [12]. Similar findings have been reported in other vascular contexts. For instance, a 90° outflow graft angle in left ventricular assist devices has been linked to elevated shear stress and prolonged residence time, both of which contribute to thrombosis [23]. Likewise, improper angulation of inferior vena cava filters during implantation may increase the risk of thrombosis and related complications [24].

Tortuosity, defined as the ratio of straight-line distance and centerline length, was identified as a key geometric factor linked to thrombus formation. Increased tortuosity indicates a longer, more winding vessel path, which can disrupt normal flow patterns and lead to greater pressure drops, both of which promote conditions favorable for thrombosis [13]. Moreover, high tortuosity can make stent graft placement more challenging, potentially leading to poor device alignment or partial apposition to the vessel wall, thereby heightening the risk of thrombotic events [25].

In this study, common cardiovascular risk factors, such as age, diabetes mellitus, hypertension, and dyslipidemia, were accounted for and showed no significant differences between patients with and without thrombotic complications. This supports the notion that local anatomical geometry, rather than systemic factors, plays a more critical role in the development of thrombus within the iliac limbs following EVAR. However, previous research has yielded mixed findings. For example, Draper et al. [7] reported a higher incidence of limb thrombosis in younger individuals and smokers, while [26] found no significant link between age and thrombus formation, though they did not assess smoking history. These inconsistencies may stem from variations in sample size, indicating that larger cohorts are necessary to produce more reliable and generalizable results. This limitation underscores the importance of future studies involving broader populations to further validate and clarify these associations.

Wu et al. [27] reported that thrombus accumulation is largely affected by the geometric design of the aortic stent graft—particularly a larger main body dimension combined with a narrower limb component and an extended main body length. These findings are consistent with our study, further emphasizing the impact of graft geometry on thrombotic complications after EVAR. In addition to thrombus formation, various studies have shown that geometric parameters also affect other EVAR-related complications, including stent graft migration and endoleaks. For example, Schuurmann et al. [15] found a significant association between increased aortic curvature and the development of type Ia endoleaks and stent migration. Similarly, Wang et al. [16] identified iliac limb migration as a key factor contributing to type Ib and type III endoleaks. Their research pointed to wide iliac artery diameters, limited fixation lengths, and overexpansion of the graft as major risk factors. Together, these studies reinforce the idea that vascular geometry is a crucial determinant not only of endoleak and migration risk, but also of thrombus formation following EVAR.

Although limb thrombosis has not been shown to significantly increase 30-day mortality (*p* > 0.05) [7], it remains a critical factor when considering the need for re-intervention and the importance of improved stent graft design, both of which directly impact patient quality of life. From this standpoint, our study offers important clinical insights. First, evaluating aortoiliac geometry before the procedure should become a standard part of EVAR planning to help identify anatomical configurations associated with higher risk. Second, intraoperative techniques may need to be adapted in cases with marked tortuosity or sharp angulation—such as employing more flexible or tapered graft limbs to better accommodate patient-specific anatomy. Third, patients with high-risk geometric profiles should receive more frequent imaging follow-up and may benefit from a more proactive antithrombotic treatment strategy after the procedure [28].

Recent studies have proven the importance of anatomical indices in the planning of EVAR. Certain anatomical features of aneurysm, including diameter, proximal neck length <10 mm, proximal neck diameter ≥30 mm, infrarenal neck angulation ≥60°, and suprarenal neck angulation ≥60°, can significantly predict perioperative mortality [29]. Artificial intelligence (AI) was also used to improve the quality of pre-procedure planning. An AI model developed by Saita et al. automatically and accurately segmented the proximal landing zone for planning thoracic endovascular aortic repair [30]. This tool allowed clinicians to perform repeatable aorta mapping, which can be helpful in cases with complex aortic anatomy.

Several limitations of this study should be noted. Its retrospective design restricts the ability to draw definitive causal conclusions, and the single-center nature of the research may limit the broader applicability of the results. While the sample size was relatively small, the use of matched control groups and within-subject comparisons strengthened the reliability of the statistical analysis. Future research using prospective, multicenter approaches, ideally incorporating CFD analysis, would offer a more in-depth understanding of the mechanisms driving thrombus formation.

## 5. Conclusions

This study highlights the significant impact of aortoiliac geometry on the development of iliac limb thrombotic complications following EVAR. Anatomical characteristics such as larger iliac diameters, decreased vessel angulation, and increased tortuosity were identified as key risk factors for post-procedural thrombosis. By recognizing and addressing these geometric risk factors, clinicians may improve the effectiveness and long-term outcomes of EVAR, highlighting the need for a more specific, anatomy-based approach to endovascular treatment.

## Figures and Tables

**Table 1:** Patient demographics.

Variables	No Thrombus (*n* = 36, 66.7%)	Thrombus (*n* = 18, 33.3%)	*p* Value
Age, years	72.11 ± 7.64	70.56 ± 9.19	0.51
Sex, male	30 (55.6)	16 (29.6)	0.59
BMI	25.24 ± 4.16	24.03 ± 3.86	0.31
Alcohol	12 (22.2)	6 (11.1)	1.0
Smoking	18 (33.3)	10 (18.5)	0.70
Hypertension	21 (38.9)	13 (24.1)	0.32
Diabetes	10 (18.5)	4 (7.4)	0.66
Atrial fibrillation	1 (1.9)	0	n/a
Dyslipidemia	9 (16.7)	9 (16.7)	0.07
Old stroke	36 (67.9)	17 (32.1)	0.15
Antithrombotic therapy			
Anti-platelet therapy	2 (3.7)		n/a
Oral antithrombotic therapy.	4 (7.4)		n/a

Note: n/a: not applicable.

**Table 2:** Comparison between the diseased and control groups.

Variables	Iliac Thrombotic Segments (*N* = 18)	Normal Iliac Segments (*N* = 90)	*p* Value *	Correlation with Thrombotic Status *p* Value (Spearman’s Rho) **
Maximum diameter, mm	17.48 ± 0.95	14.14 ± 0.62	**0.006**	**0.002 (0.362)**
Minimum diameter, mm	9.47 ± 0.78	9.88 ± 0.59	0.682	0.201 (−0.380)
Maximum sectional area, mm^2^	195.81 (65.11–301.12)	147.4 (110.87–258.13)	0.093	0.395 (0.102)
Minimum sectional area, mm^2^	67.01 (47.18–84.24)	76.09 (61.89–89.12)	0.339	0.969 (−0.005)
Graft limb angle, degree	117.52 ± 5.61	148.54 ± 4.31	**<0.001**	**0.004 (−0.332)**
Aortoiliac angle, degree	123.48 ± 4.66	141.96 ± 4.76	**0.009**	**0.047 (−0.225)**
Graft limb tortuosity	0.2 ± 0.03	0.12 ± 0.02	**0.021**	**0.011 (0.189)**

Note: * Independent sample *t*-test (normally distributed variables) and Mann–Whitney U test (non-normally distributed variables). ** Spearman’s test. Bold font indicates statistical significance (*p* < 0.05).

**Table 3:** Paired analysis conducted between normal and thrombosed iliac segments within the same patient in thrombotic group (*n* = 18) and between right and left iliac segments within the same patient in control group (*n* = 36).

Variable	Thrombotic Group (*n* = 18)		Control Group (*n* = 36)	
	*p* Value (Paired *t*-Test)	Mean Difference Thrombus Minus Normal	*p* Value (Paired *t*-Test)	Mean Difference: Right Minus Left
Maximum diameter, mm	**0.012**	3.339	0.12	1.8
Minimum diameter, mm	0.404	−0.406	0.78	−1.2
Maximum sectional area, mm^2^	0.088	44.116	**0.042**	10.1
Minimum sectional area, mm^2^	0.794	−13.211	0.94	−8.2
Graft limb angle, degree	**0.001**	−31.022	0.25	7.2
Aortoiliac angle, degree	**0.005**	−18.472	0.48	−8.2
Graft limb tortuosity	**0.027**	0.077	0.98	0.08

Note: Bold font indicates statistical significance (*p* < 0.05).

## Data Availability

The datasets generated or analyzed during the study are available from the corresponding author upon reasonable request.

## Abbreviations

The following abbreviations are used in this manuscript:

EVAR	Endovascular aneurysm repair
AAA	Abdominal aortic aneurysms
CTA	Computed tomography angiography
GLA	Graft limb angle
AIA	Aortoiliac angle
GLT	Graft limb tortuosity
rho	Spearman correlation coefficient
CFD	Computational fluid dynamics
AI	Artificial intelligence

## Appendix A

**Table A1:** Comparison between our present study and a previous one in terms of iliac thrombosis risk following EVAR.

	Previous Study [12]	Present Study
Inclusion criteria	Post-EVAR thrombosis of aorta and/or iliac artery	Only thrombosis of iliac artery
Measurements of vessel dimension	Circumferences and sectional areas of the aorta and iliac components	Diameter and cross-sectional area of iliac components
Angles measurement	Bifurcation angle: angle between two iliac arteries	Aortoiliac angle (AIA): angle between aorta and iliac artery. Graft limb angle (GLA): angle between iliac stent and non-stent segments
Tortuosity measurement	Proximal aorta to distal iliac stent segment	3 cm above and below iliac stent distal end
Results for iliac thrombosis	Statistically significant difference in iliac circumference sectional area.	Statistically significant difference in maximal diameter, GLA, AIA, and iliac tortuosity
